# “Anyone Else Struggling with Work-Genocide Balance?” Exploring the Psychological and Social Impact of Collective Annihilation in Gaza

**DOI:** 10.1007/s11013-025-09953-0

**Published:** 2025-10-22

**Authors:** Federica Cavazzoni, Ala’ Mustafa, Cindy Sousa, Ola H. Abuward, Mona Ameen Nofal, Guido Veronese

**Affiliations:** 1 University of Milano-Bicocca, Piazza dell’Ateneo Nuovo, 1, 20126 Milan, Italy; 2 An-Najah National University, Nablus, Palestine; 3 Bryn Mawr College, Philadelphia, USA; 4 Gaza Community Mental Health Program, Gaza City, Gaza, Palestine

**Keywords:** Colonial violence, Mental health, Trauma, Community resilience, Dehumanization

## Abstract

This study investigates the psychological and social impact of the ongoing colonial violence in Gaza, both on the local population and on distant witnesses. Drawing on 30 testimonies collected between November 2023 and June 2024, the research captures the lived experiences from two distinct groups: 15 Palestinian participants living in the Gaza Strip, and 15 mental health professionals (including psychotherapists and academics) based in Europe. Using phenomenological and thematic analysis, the findings reveal the profound effects of genocidal violence on mental health, emotional resilience, and meaning-making processes. For Palestinians, daily exposure of bombardment, displacement, and systemic dehumanization undermines personal and collective agency, resulting in emotional numbness, anger, and alienation. Witnesses from European contexts reported helplessness, disorientation, and moral injury, often struggling with their perceived complicity in global systems of oppression. The study challenges conventional trauma frameworks, emphasizing the need to conceptualize colonial trauma as continuous, collective, and politically rooted. It argues for integrating social and political dimensions into psychological approaches to trauma, and highlight the ethical and political importance of bearing witness. Ultimately, the paper calls for a transnational process of collective healing and solidarity, aimed at dismantling the structural foundations of violence and dehumanization.

## Introduction

“Anyone else struggling with work-genocide balance?” Exploring the psychological and social impact of collective annihilation in Gaza.

I always think of Gaza. Yes, it’s true; I get up, go out, and do my things, but I always think of Gaza. The more things I do, the more I think of Gaza. If I turn on the tap, I think of Gaza, which has no water; if my son has a fever, I think of Gaza, which has no medicine; if there is a tremor, I think that in Gaza, bombs explode.

I always think of Gaza.

Everything I do serves to distract me, yet it doesn’t help because I know that behind it all is Gaza.

“Try writing; maybe it will help you find clarity.”

And no, I don’t want to find clarity. Why should I force myself into rigor? Is it the only position to take while the genocide of the Palestinians is being carried out? While their lives, their existence, are being turned into a desert, besieged like the high walls of Ilion? (Valeria Parrella)

She wonders if I take notes in Arabic.

I shake my head, no

She pays attention to the remains of tears

Focuses carefully

She puts her hand on my wrist

And she asks

Are you okay?

In my head, I’m not okay at all

No one should be okay

But I shake my head in agreement and put a fake smile on my face

The Greek researcher sits

Her Italian colleague continues talking about their joint research

Researchers continue to present their research

And I keep clapping

And the world continues its rotation

I wish it would realize

Even for a second

That it must stop and cry blood over the ugliness of its children” (Ala’ Mustafa)

If the first of these two testimonies is taken from an Italian newspaper some twenty days after the start of this genocidal attack on Gaza, Palestine, the second is instead from one of the authors of this work, in a moment of daily life within these—at the time of writing (August 2024)—305 days of genocide.^1^ It is a fragment of a small and powerful poem written during a day of ‘ordinary’ research work while experiencing the internal fragmentation of those who witness the genocide of a people or their people.

“How is your work-genocide balance?” a colleague asked us one November morning. She asks in a WhatsApp group where some participants are in a part of the world observing Gaza from afar, scrolling through Instagram between images of vacations in the Maldives and pictures of blood on sacks of flour. How do these images meet within us, and how do they find space in our routine? She asks this in a group where some participants are instead displaced to the south of Gaza, sheltered in a school, after witnessing the destruction of their homes, their universities, hospitals, and their cities, trapped within the alternate space-time of colonial violence (Ihmoud, 2023), waiting for death while fighting for life. A few days before that message, one of the displaced colleagues had updated us after being without electricity and internet for days. She informed us that she had survived a long night of bloodshed and had listened to the radio throughout the night. The radio spoke of some protests in Jordan in solidarity with the Palestinian population. “It was imperative”—she writes to us—“because from here, it was impossible to know if people there and colleagues were talking about what was happening. It was impossible to know if the world had stopped to demand an end to what was happening or if silence reigned instead.”

As academics and psychologists—like the authors of this work—we recognize that the impact of war extends beyond the physical destruction of the world; it also devastates the social fabric that shapes one’s history, identity, and values (Summerfield, 2000). While much of the psychological and psychiatric literature focuses on the intrapsychic wounds caused by displacements and cultural genocides of colonialism, there has been less attention given to the psychological effects on those who are complicit in oppressive structures, on those who have benefited from colonialism. Does silence prevail, with passive observers taking the lead, or is there space for those who subvert this ‘illness of normality’ (Gruen, 1999)?

In this work, which consists of a reflective discussion on a series of testimonies collected inside and outside Gaza over these 10 months among Palestinians living in Gaza and Italians living in Europe, we aim to explore how this genocide impacts daily life—for those in Gaza struggling to survive, and for those outside Gaza witnessing it. Specifically, we ask: How does this genocide impact mental health, in particular as it challenges the meaning and sense of coherence people experience in the world? For those outside Gaza, we considered how psychologists process all they witness, confronting the precariousness and evidence of the double standard of morality, as well as the values upon which we have built our foundations of our societies and lives.

Our theoretical framework draws from post-colonial and feminist perspectives, which allows us to critically interrogate the dynamic of power, oppression, and solidarity. These framework helps us to provide the lens through which we understand the comparative experiences of the testimonies, and enables us to analyze how witnessing suffering can generate both psychological dissonance and a sense of moral urgency, guiding our exploration of solidarity in the context of genocidal violence. In line with this framework, as we interpreted the data, we interrogated the nature of pragmatic solidarity (Farmer, 2004), when some colleagues are unescapably located within a genocidal project, and others can only bear witness from afar. We also examined how bonds among mental health researchers offer personal and professional solace, as well as a guide forward, when time seems to stand still in the suffering (Lykes & Sibley, 2014). Finally, especially for those outside of the tragedy, we reflect on how psychologists may best bear witness, refusing to remain in silence, and mobilizing their practice and research to accompany those directly affected and to address the structural iniquities that perpetuate such violence (Abrego, 2024; Orbinski et al., 2007).

## The Context

### ‘Invasion Is a Structure, Not an Event’—Settler Colonialism and Palestine

The Gaza Strip is a small coastal enclave, bordered by the Mediterranean Sea to the west, Egypt to the south, and Israel to the north and east, although it has no legally defined borders. It is part of the so-called occupied Palestinian territories (oPts) (as designated by the United Nations since the Israeli occupation of ‘67), along with the West Bank and East Jerusalem, to which it is not geographically connected. With approximately 2.2 million inhabitants in 2022 (including 1.1 million children), it is widely recognized as one of the most densely populated places in the world. Living conditions have long been impossible and unlivable due to the siege imposed by the Israeli occupation, which has affected all Palestine for over 50 years. In 2012 Chomsky described Gaza as ‘the world’s largest open-air prison’ and, in 2020, the United Nations declared it uninhabitable.

In Palestine, Israel’s expansionist policies have escalated every year since 1948, through the construction of settlements, house demolitions, and land confiscation (Salamanca, 2014; Shihade, 2012). Palestinians are deprived of every fundamental right and forced to live without predictability or continuity, whether social, spatial, or economic (Itani et al., 2017; Shalhoub-Kevorkian & Odej, 2018). Their freedom, physical integrity, and ability to work, acquire property, move, and plan for the future are all dependent on Israeli authority and control (Illouz, 2014). Policies such as curfews, daily incursions, movement restrictions, arbitrary checkpoint closures, and systemic humiliation compound the psychological, economic, physical, and cultural oppression (Barron & Abdallah, 2015). These Israeli state policies of occupation and oppression must be understood by moving away from the conventionally adopted ‘lens of exceptionalism’ (Busbridge, 2018) and instead through the lens of settler colonialism studies (Busbridge, 2018; Greenstein, 2019; Veronese et al., 2022). Unlike colonialism driven primarily by economic exploitation, settler colonialism operates through the logic of land acquisition and the elimination of Indigenous populations, not only as individuals but, crucially, as peoples with sovereign status and claims (Wispelwey et al., 2023; Veracini, 2011). This logic enables the replacement of Indigenous communities with settler populations, who justify land dispossession through racialized narratives of superiority, a hallmark of Western modernity and racial capitalism (Wispelwey et al., 2023).

This reality extents across all Palestine, but daily life in the Gaza Strip is even more dire. Following Hamas’ electoral victory in 2006, Israel has imposed a strict blockade on Gaza, controlling and severely restricting the movement of people and goods, including essential supplies. This has led to the total complete collapse of the Strip’s economy, turning residents dependent on international aid (B’Tselem, 2017). In 2018, the United Nations Relief and Works Agency for Palestine Refugees in the Middle East (UNRWA) showed that 95% of Gaza’s inhabitants did not have access to clean water—following the destruction of water purification plants by Israel—and electricity remained at minimal levels (between 4 and 12 h a day), affecting every aspect of daily life (e.g., healthcare services, agricultural sectors) (UNRWA, 2018). Well before October 7, Gaza had been described by B’Tselem—an Israeli human rights organization—as ‘a humanitarian disaster entirely caused by man, the direct result of official Israeli policies’.

As in other situations of settler colonialism, the Israeli occupation is not only a political issue but also a mental health issue (Jabr, 2019; Hammoudeh et al., 2013). Ethno-political violence by Israel against Palestinians has been persistent for over three generations, subjecting both adults and children to daily humiliations, violence, traumatic losses, and traumatized parenting, which are hardly captured by post-traumatic stress disorder diagnoses (Barron & Abdallah, 2015; Giacaman, 2018). Alongside concerns for personal safety and that of their significant others, difficulties related to access to humanitarian care, education, and movement have been linked to a substantial increase in stress, anger, frustration, and fear (Bryant-Davis & Ocampo, 2005; Cummings et al., 2017). Moreover, humiliations, traumas, and injustices wound the individual and collective psyche, eroding the moral and social fabric of entire communities. Colonial trauma is a complex, collective, continuous, and cumulative trauma. By its very nature, it requires a re-evaluation of the dominant biomedical model of trauma to avoid distorting social suffering into an individual psychobiological disorder (Marshall & Sousa, 2017; Mitchell, 2019; Nguyen-Gillham et al., 2008). Colonial violence disrupts one’s sense of meaning and coherence in the world, necessitating an approach that acknowledges its political and historical dimensions in any effort toward healing and meaning-making. Mental health professionals working to uncover the impacts of such violence and suffering—and to understand how people psychologically endure it—are themselves constantly ordering and re-ordering their mental and emotional schemas. In the face of situations so cruel that they evade comprehensibility, scholar-practitioners try to derive not only personal, but also scholarly, meaning and coherence. In moments of acute suffering, this meaning often escapes even the most seasoned mental health professional. In these moments, generative, collective communities of scholarship offer a jointly created “third space” (Bhabha, 1999) where work intersects with “real life,” and conversation, joint documentation, and scholarly sense-making to offer not only comfort, but also an emotional/intellectual path forward.

### The Ongoing Genocide in Gaza

The Gaza Strip has been subjected to several military attacks by Israel from 2007 to the present (2008/2009, 2011, 2014, 2021). Still, the one that began following the Hamas militants’ attack on October 7, which killed approximately 1200 people and took 240 hostages, is the most devastating and enduring, associated with a complete siege on all land and sea borders.

At the time of writing,^2^ the brutal Israeli campaign is estimated to have killed more than 186,000 people within the Gaza Strip, equivalent to 7.9% of the Strip’s total population, with an estimated 70% being women and children (Khatib et al., 2024). Over 1,700,000 people have been displaced (OCHA, 2024), and bombs and military targets have damaged and destroyed between 60 and 80% of all commercial and residential buildings and activities in Gaza. United Nations experts have tweeted that today 80% of those facing famine globally are inhabitants of Gaza. 19,000 children have been orphaned due to the killing of their caregivers. Every day, 10 children in Gaza lose a limb, amputations are at times done without anesthesia, and every wound is at risk of infection (Save the Children, 2024).

As with everything in this historical moment, we have had access to images and videos documenting every day of the Israeli military aggression over these 10 months: attacks on hospitals, journalists, schools, and universities. Indiscriminate bombings, killings of civilians, bombings of refugee camps. Some of these documentations of extreme violence have come not only from survivors and journalists but also from Israeli soldiers themselves, who have filmed and photographed their own crimes and shared the images on social medial. Of Gaza’s 12 universities (University of Palestine; Palestine Technical College; Israa University; Islamic University of Gaza; Al Azhar University; Gaza University; Al-Aqsa University; University College of Applied Sciences; University College of Science and Technology; Palestine College of Nursing; Arab College of Applied Sciences; Al-Quds Open University; Hassan University), none remain standing today, leading to what was defined—as early as 2008—as ‘scholasticide’ (Nabulsi, 2009) or ‘sophicide’ (Fúnez-Flores, 2024), a deliberate uprooting of indigenous knowledge anchored in the land and its custodians (Palestinian Feminist Collective, 2024). Sophicide and scholasticide refer not only to the physical destruction of institutions and educational resources but also to the erasure of Palestinian history, archives, and epistemologies (Fúnez-Flores, 2024).

Before October 7, leaving the Gaza Strip was already particularly challenging. One had to obtain an Israeli visa to exit through the Erez crossing, which was practically impossible for a Palestinian citizen of Gaza. It was possible to leave through the Rafah crossing with Egypt when it was open, though there were considerable queues and difficulties (OCHA, 2022). Since October 7, exiting through Erez has become impossible, and the people who managed to leave the strip from October to May 2024 did so through the Rafah crossing via a complicated, lengthy, and costly waiting list process. Leaving Gaza costs about $5000 per person and $2500 per child. Since May 7, 2024, Israel has taken control of the Rafah crossing and closed it, declaring it impossible for anyone to leave Gaza while the invasion continues by air and land.

For over 10 months, images of the ongoing situation have reached those outside of Gaza, revealing to the outside world the complete lack of food, water, and medicine in Gaza. These images show humanitarian aid trucks blocked at the entrance by Israel, adults and children dying of malnutrition, mass graves, tortured prisoners, surgeries performed without anesthesia, and childbirths without water. Everyone in Gaza is in desperate need of mental health support, as are those of us who find these atrocities unbearable even from the safety of our homes—in a continuous state of shock and horror at the prolonged nature of this terrible genocide. In this work, we have aimed to investigate how this genocide impacts both the daily lives for those under the bombs in Gaza struggling to survive and for those witnessing it from afar, and our professional sensibilities and commitments, as engaged mental health practitioner-scholars.

## The Study

The group of authors behind this article has known each other for several years, some more closely than others, through their academic work and solidarity activism in and for Palestine. They all work in the fields of psychology, human rights, and mental health. Two of the authors are young researchers from Gaza who have either lived through—or are currently living through—this genocide with their own lives and bodies. One was displaced, her house was destroyed, her family separated, and she managed to flee Gaza in April 2024. The other has endured multiple forced displacements and remains trapped in northern Gaza, where famine is most severe. The other three authors—a PhD student from the West Bank who is studying in Europe, two academics from a European country (a post-doc and an associate professor), and one associate professor from the US—constantly grapple with the unfolding genocide in Gaza. They balance grief, guilt, and existential anxiety with the demands of productivity and the expectations of living an ‘ordinary life’. Though we have a collective body of work on the topics of political violence, social suffering, and survival that spans decades, the heavy weight of personal ties to Gaza makes the experience of feeling like we are currently helpless observers of genocide unbearable. The ever-present fear looms that the familiar violence from military raids could escalate into full-scale genocide all over Palestine at any moment.^3^ In the face of this existential despair, that is all at once personal and professional, we have come together to write this work as a collective journey, in an attempt to make sense of the disorientation, pain, and guilt we share.

The chosen perspective is informed by critical, decolonial theories and liberation psychology, which are fundamental for addressing mental health in contexts of war and colonial oppression like Palestine. Liberation psychology is a movement that emerged in the 1970s in Latin America, focusing heavily on the impact of social and historical contexts on mental health, questioning the impartiality of research and clinical practice. Its goal is to understand the distress of oppressed communities through a structural lens, viewing this distress as a normal reaction to abnormal and violent living conditions (Comas-Díaz & Torres Rivera, 2020; Makkawi, 2017; Marshall & Sousa, 2017; Montero & Sonn, 2009). Like other psychological approaches, liberation psychology describes psychological suffering, analyzing its deep-rooted causes and proposing healing practices. However, it specifically emphasizes the connections between this suffering and the social, economic, and political contexts in which individuals live. It aims to understand the interdependence between the intrapsychic, interpersonal, community, economic, and environmental contributions to the structure of experience and the larger frames of culture and history in which these are embedded. Thus, it is a crucial approach for recognizing the psychological wounds caused by oppression, and for crafting collective responses to these wounds that heal both individuals and societies (Martín-Baró, 1994). From the standpoint of engaged research, our approach considers the dynamic ways in which scholars can, together, experience, witness, and study social suffering and, also together, in community, find ways to utilize research relationships and tasks to build personal and professional survival (Abrego, 2024; Rosales et al., 2024). The study was conducted in line with APA ethical guidelines (APA, 2016) and had been approved by Milano-Bicocca University IRB (N.776)

### Participants, Instruments, and Procedures

Together, we analyzed and discussed a series of testimonies (N=30) collected by the authors themselves during these ten months of genocide in Gaza. Fifteen testimonies come from individuals in and from the Gaza Strip, aged between 20 and 56 years (73% women). These participants were recruited through convenience sampling, based on one of the authors’ capacity to move within Gaza and connect with individuals despite the extreme conditions. They worked in a variety of sectors and civil society associations. Approximately twice the number of individuals were initially approached; some declined to participate due to logistical difficulties or lack of desire to share their personal experiences in such a context. The rational for including participants from Gaza was self-evident: to center the lived experiences of those directly subjected to the genocidal violence unfolding in real time. The remaining 15 testimonies were collected in a European context, specifically in the country of two of the authors. All these participants in this group were Italian citizens. This sample was also selected through convenience sampling and included colleagues in the field of clinical psychology—both academics and psychotherapists—aged between 27 and 60 years (67% women). These participants were selected based on their previously expressed emotional and professional concerns in relation to the situation in Gaza, despite not having personal or professional connection with Palestinians or involvement in work on Palestine. As with the Gaza group, roughly twice as many individuals were initially contacted; those who declined cited limited availability or discomfort in addressing such emotionally charged topics. The decision to include both groups—individuals experiencing the genocide firsthand and professionals observing it from afar—was intentional. Our aim was to explore not only the direct impact of genocidal violence but also how such violence reverberates globally, shaping the emotional and ethic lives of distant witnesses. All participants received an informed consent form that outlined the aims and modalities of the study. in line with the constraints of the context—particularly inside Gaza—verbal informed consent was obtained from all participants, including explicit consent to audio-record the conversations.

The testimonies were collected through semi-structured interviews aimed at investigating the daily experience during this genocide. More specifically, for the present work, we focused on the sections where the discussions revolved around their experiences of displacement, bombing, and their ability to make sense of what is happening. In the interviews conducted in Italy, we focused on the sections where they reflected on how the ongoing genocide impacted their daily lives and mental health. The interviews were collected from early November 2023 to mid-June 2024 by two authors in the context of Gaza and one author in the European context. The interviews ranged from about 25 to 60 min and were audiotaped, transcribed, and—where needed—translated from Arabic or Italian to English by two authors. They were back-translated into Arabic or Italian and compared against the original interviews to ensure the accuracy of the translations.

A reflective and experiential approach was adopted to guide this study, which was considered the best way to conduct an in-depth examination of the opinions, doubts, and perspectives on these issues (Clarke & Braun, 2013; Haverkamp & Young, 2007). All transcripts were analyzed through a phenomenological and thematic analysis (PTA), proceeding through the six stages outlined by Clarke and Braun (2013). The analysis was carried out in three main steps. Three authors of this work independently read all the transcript and identified main themes relevant to the aim of this work. The authors met in discussion sessions to review the identified areas jointly, observing how their assumptions and viewpoints influenced the interpretation of the words. Nvivo 12 qualitative software was used to facilitate data management and discussion among the authors. Some themes were merged in this step, forming overarching categories with sub-themes. Finally, authors reanalyzed the interviews with the final themes and then discussed their agreement and the meaning of these themes.

### Findings

What follows is a textured account of how participants made sense of the ongoing genocide, revealing a spectrum of emotions, contradictions, and acts of meaning-making. The findings are presented through three overarching themes, each shedding light on different facets of lived experience—how participants endure, resist, and make meaning in the face of sustained violence. Rather than aiming for representativeness or quantification, we focused on capturing the breadth and depth of participants’ narratives (Cavazzoni et al., 2025; Marshall, 1996).

### The Ordinary Israeli Brutality and Madness

What does daily life look like in the Gaza Strip after nearly a year of relentless bombardment, isolation, and deprivation? This section explores the lived experience of people enduring brutal colonial violence, where survival is uncertain and the fabric of everyday life is shattered by constant destruction.

My mother, my sisters, and I were trapped under the rubble for 45 minutes when they bombed the neighborhood. It took me a while to process what had happened. It was helpful to stay awake; it seemed like there was no oxygen dust in my eyes, nose, and mouth. I tried to spit it [the rubble] out, but the wall was very close to my mouth, so I had to swallow the dust; it was the only way to keep breathing... After that, I don’t remember anything. I woke up, and a week had already passed. They say I was delirious at first. My skull was fractured; they thought I would lose some of my functionalities or wake up with amnesia. I was also somewhat catatonic. I lost 8 units of blood. When I woke up, I started asking about my family; no one knew anything about them. I heard from someone that my mother was a martyr. When I found out, I went back to intensive care and fainted for another 3 days. (p.3, W, 22y.o., Rafah, Gaza)

The testimonies describe the terror and despair of being trapped under the rubble, unable to breathe amidst debris and dust. They highlight how physical pain blends with the psychological trauma connected to the total loss of a sense of safety for oneself and one’s family. Every day is filled with mourning, pain, and isolation.

The hardest moment was not being under the rubble; it was waking up injured and being told that none of your family is here. I felt the physical pain of that; I felt my internal bleeding as if it were coming from my soul. It was a moment I will never forget. They ask me how I survived. Do you think I survived? (p.1, W, 24y.o., Rafah, Gaza)

I am full of fear. We are still paying off the debts for the house we bought, which we had to pay off by 2035. Now that house has been destroyed for fun. They used it as a military base, then burned it. I lost my house for nothing. We own nothing now, so there is nothing to sell. There is no one to rely on. I can’t think about the future; I am still trying to grasp reality. I can only say that I am truly afraid of the future. I am still processing. (p.13, W, 30y.o., displaced in Rafah, Gaza)

Many testimonies bring up the theme of survival, and the complex dynamics wherein physical survival does not necessarily mean one can survive psychologically. The past has been destroyed, burned, demolished. The sense of the future is lost, and the present is unbearable. The accounts introduce us to experiences of continuous displacement, forcing people to move from one place to another amidst fear and bombings, losing contact with their community and family.

And then we arrived at a school. In the school, it’s another story of struggle, deprivation, and suffering. Life in the school does not resemble life at home at all. It’s a life similar to being on the street. There is no clarity, no calm. Inside the classroom, you live with 40 people. Outside, nearly 2500. In school, you don’t just experience the genocide of bombs. (p.5, W, 27y.o., displaced in Rafah, Gaza)

The daily battles faced by the people of Gaza are endless, even when they find a place to take refuge, ‘similar to being on the street,’ insecure and unstable. They are unable to protect themselves from diseases, cold, and hunger. You do not have a sense of privacy or feel you are in a confidential place. Everything you say is heard by the people next to you. For those taking refuge in schools, it also meant thousands of women sharing 10 toilets, and water was usually available for only two hours a day.

This narrative went on to detail the multiple meanings of war, all at once:

There are many wars here. There is the war of hunger, the war of epidemics and diseases, the war of cold, the war of food and water, the war of menstrual cycles, the war of giving birth in the school, the war of calming oneself in these conditions, the war of mourning. The war of being in the persistent cycle of fear of losing someone from your family. All these are wars. When we work on the impact of war, do we consider this? (p.2, W, 32y.o., displaced in Rafah, Gaza)

This narrative explains plainly what years of research document, that wars and associated political violence encompass every aspect of living under the violence—that it goes beyond simply one bombing or one event, that it permeates all activities of living. The daily misery caused by attacks are also detailed below:

We lack food and water. Each of us has two dates for breakfast, a cheese sandwich for lunch, and a cup of milk for dinner if there is milk and water to dilute it. Otherwise, we have another two dates. I had my period a few days ago and couldn’t wash because there is no water, no sanitary pads, nothing. (p.6, W, 55y.o., displaced in Rafah, Gaza)

You don’t know how war affects your body. We have eaten barley, we have eaten pigeon feed, and we have eaten things not fit for human consumption... we don’t feel like human beings. We feel like animals, like donkeys. No one looks at you as a respected human being, nobody out there gives you anything, asks how you are, or asks about your health... let’s not kid ourselves. (p.8, M, 27y.o., North Gaza)

There is constant exposure to danger, fear, loss, and the struggle to meet basic needs, creating cumulative trauma on the psyche of individuals and the community. Collective traumas dehumanize: here, the passage details how the war causes a catastrophic disruption in our sense of personal and communal identity, interrupting the ways of thinking, speaking, acting, and relating that we previously used to make sense of the world. This disconnection deprives us of the usual reference points through which we build our understanding of reality, leaving us lost, disoriented, and fragmented in our perception of self-as-human, as well as our place in community and the surrounding world.

The testimonies here report being stripped of everything: their safety, their stability, and above all, their dignity, providing a dramatic snapshot of the psychological and physical devastation caused by the war. The loss of self, surrender, and emotional isolation are consequences beyond physical wounds.

War can turn you into a skeleton, another person, or someone you don’t recognize. War can leave you with nothing, literally nothing. You end up not even being able to recognize and find yourself anymore. (p.11, M, 50y.o., Rafah, Gaza)

If I had to describe my mental state? I can sum it up better; I only need three words, and I’ll say it in English, I GAVE UP. I don’t care about anything anymore, I don’t watch the news, I have no passion for anything, I have no plans for the future, I’m too exhausted, let things happen as they must, I’m just living day by day. (p.7, W, 24y.o., displaced in Khan Younis, Gaza)

I am mentally exhausted. My energy is depleted. After 168 days of genocide, I am now going through silence. I look at corpses, destruction, the wounded, and bodies torn apart, and I feel sadly silent. I don’t write, and I don’t talk about it. I no longer talk to anyone [colleagues outside Gaza] about what I see because they are all busy with their lives. Our voice has become annoying and burdensome to the world. (p.2, W, 32y.o., displaced to Rafah, Gaza)

The testimonies speak of the exhaustion that people feel after 10 months of heroic struggle for life amid death. This level of overkill (Jabr, 2019), in addition to the obvious physical consequences, produces estrangement from self and others—a pervasive loss of identity and collective belonging.. Perhaps most painful to witness in this narrative is the sense that this estrangement, this withdrawal and silence, is preferred by the world. The resignation reflected in these passage reflect alienation of those who suffer from the rest of humanity, including distance and isolation from colleagues.

### (No) Business as Usual

The second theme, on the other hand, includes reflections mostly found within the testimonies collected outside Gaza from those observing Gaza. It highlights the angst of the continuity of daily life as people experience rupture with the values and conceptions of the world that previously guided everyday existence.

How do we sleep at night while all this is happening? How do other people sleep at night? (p.17, W, 42y.o., Europe)

I am living with an obsessive question. How will the people in Gaza overcome the awareness of their torture and globally visible limbs, knowing that no power has done anything to stop it? How could they (and we) still have faith in humanity? (p.22, M, 37y.o., Europe)

The testimonies describe a strenuous attempt to reconcile one’s grasp of morality and meaning while being constantly exposed to the events occurring in Gaza and the genocidal practices supported or not hindered and denounced by their governments and colleagues. This state of cognitive dissonance—between the devastation of what is observed and the ‘business as usual’ that is lived—leads to experiences of disorientation, bewilderment, anger, frustration, and powerlessness that permeate every aspect of life. These narratives demonstrate the angst of witnessing cruel violence and destruction and feelings of overwhelm, both practical and existential:

I am trying to manage my anger. I am overwhelmed by the pain. My stomach hurts. How do I explain that burning children alive in tents is wrong? Who doesn’t know that? (p.27, W, 35y.o., Europe)

How do I maintain faith in humanity, function day to day, and not fall into a pit of depression while watching a genocide unfold and knowing that none of our governments truly denounce it? How do I not feel responsible? We are part of that side of the world that supports these colonial policies. (p.27, W, 35y.o., Europe)

I never thought the world was a fair and just place. But I admit I never thought we could witness what Israel has been doing for over five months and know that no European government will move to stop it. (p.30, M, 44y.o., Europe)

These reflections reveal the emotional and psychological impact of witnessing atrocities and oppression, especially when one recognizes belonging to the group that perpetuates or supports such injustices. A profound crisis of trust in oneself and the power systems to which one belongs, which are complicit, exacerbating the sense of guilt and powerlessness, is discussed. The awareness of the complicity of one’s government and academic and psychological institutions in the brutal and violent oppression observed in Gaza seems to undermine the moral and ethical foundations upon which personal and collective identity is based, leading to an experience of profound disillusionment and alienation.

The testimonies raise a crucial question that generates deep discussion: is striving to maintain a semblance of normality during a genocide a sign of mental health? The ability to continue with daily activities and maintain stability is also referred to as an act of resilience and psychological adaptation. On the other hand, being indifferent and unchanged in the face of such atrocities can be considered symptoms of a very troubling pathology.

Who is not well? What does it mean to be well? Am I well if I can’t get my mind off it, or am I well if I can’t keep my mind on it? What kind of dissociation must we activate to ‘be well’? (p.18, W, 51y.o., Europe)

We already knew, but we have faced even more the awareness of the potential barbarity of which our civilized West is capable and responsible. (p.17, W, 42y.o., Europe)

This loss of meaning and trust does not spare the various institutions and orders the interviewees are part of in their daily lives. From this perspective, they question the silence within their institutions—academic and psychotherapeutic—understood as silence to avoid dealing with the ethical failure of their profession.

Are we so silent because we cannot recognize the pain within ourselves? It is not easy to deal with our positions as perpetrators, beneficiaries, and spectators without falling into the deadly narcissism of white guilt? (p.22, M, 37y.o., Europe)

We were bewildered by the silence of our institutions. Is it silence or silencing? I don’t know, but the feeling is one of an atmosphere of self-censorship to avoid controversy. What is the controversy? How do we explain the silence in therapeutic and analytical communities, accustomed to depth and thinking the unthinkable? (p.18, W, 51y.o., Europe)

Reflections on silence are manifold. On the one hand, it is described as a silence due to a sense of powerlessness, accentuated by the fear of falling into self-indulgent narratives connected to one’s guilt without concrete actions for possible change. From this perspective, they emphasize recognizing one’s responsibilities and privileges—both individual and collective—as a necessary step towards active engagement. On the other hand, the disparity in reactions—such as the contemporary response to Ukraine—is highlighted, a conflict that received more attention, action, and solidarity compared to what happened in Gaza. If silence could be explained in terms of a fear of speaking out on ‘political’ issues for fear of being perceived as less ‘objective and neutral’ at the expense of ethical and moral commitment, this comparison invalidates that possibility.

I saw a double standard. ... The world helped the Ukrainians, but no one helped us. No one even helped me evacuate my mother and sister from Gaza. (p.25, W, 29y.o., Europe)

I wonder why our profession remains silent. It wasn’t for other wars, it wasn’t for Ukraine, we got involved. What is the concern about speaking out? Being considered less clinical or less academic? (p.27, W, 35y.o., Europe)

You felt the difference within yourself with every news report. The West is outraged by the death of a child in Ukraine outraged by the bombing of a hospital in Ukraine. Rightly so. About Gaza? No one is outraged. Lives are not valued equally. (p.29, W, 27y.o., Europe)

The testimonies discuss the double standard in the international response to the two ongoing conflicts, raising cultural, economic, political, and racial aspects that influence the perception of the value of human lives.

## Countering Silence, Isolation, and Alienation

### The Importance of Community

The testimonies also discuss modes of survival and resistance to the loss of every aspect of the self and the sense of isolation and alienation perceived during these 10 months.

Firstly, from within Gaza, the importance of social support and community—however fragmented due to continuous displacements—is highlighted as crucial for surviving colonial violence.

The people around me helped me survive. Al-Khansaa said “If it were not for the many people around me crying, I would have killed myself”. We sat with people and their tragedies surrounding us. We consoled each other, we talked, we stuck together. (p.15, W, 54 y.o, displaced to Rafah, Gaza)

What kept me patient and alive is my belief that life is still long and full of possibilities. I still have opportunities to build a life better than the one I lost, and that I don’t want to let my family down. Their faith in me keeps me going. Their presence keeps me going. Our community keeps me alive (p.7, W, 24y.o, displaced in Khan Younis)

Secondly, the importance of feeling part of a community in the face of emotional isolation, to share the pain and outrage over the suffering in Gaza, is emphasized.

It is difficult to continue with your routine. I feel like I can’t talk to anyone about it. It’s strange; other times, in the face of terrible wars, you felt externally like a community with which to share it. With Ukraine, for example, this happened. With colleagues, we got involved, we wrote, we helped. We moved. There was a collective experience. Here? Nothing is mentionable. Mourning and loss need a collective aspect to be lived. I don’t have a community with which I can do this. (p.30, M, 44y.o., Europe)

It is difficult to go on with work, mainly because I feel alone. I think about Gaza, about what is happening, about what we are complicit in. But I feel alone in worrying about it. I need to find a community where I can express it. Where we can grieve together, get angry together, activate together. (p.27, W, 35y.o., Europe)

The importance of collective support in mourning and anger is discussed. The lack of such a community amplifies the sense of isolation and powerlessness, hindering a collective response to the crisis. In line with Abdaljawad Omar’s question, ‘Can the Palestinians mourn?’, the testimonies question whether it is possible to mourn the Palestinians. Just as a funeral wake represents a communal act, the testimonies highlight the need to mourn collectively to acknowledge the trauma and the actions needed to seek justice. The militancy of mourning—writes Jabr (2019)—is not a lament but a demand for recognition and action. Without a shared social awareness, the mourning process is hindered, equivalent to saying there is no peace without justice.

### Community as a Place for Shared Meaning

Another central aspect discussed is the need to trace back a sense of meaning (Frankl, 1985) to counter the feeling of disorientation and despair and to reconnect with reality.

I feel like I am in a moral conundrum. Nothing makes sense; I need to find meaning again. (p.30, M, 44y.o., Europe)

How are we incapable of making sense of reality without distorting it? Is it vital to deny, ignore, or silence any recognition of the Palestinian people’s agony? All this hypocrisy and falsehood has driven me crazy in these months. Finding a community with which I can share this sense of madness and alienation has allowed me to breathe again. And to think about taking action. (p.27, W, 35y.o., Europe)

The moral contradictions of individuals, says Hanna Arendt (1962), can be accentuated by the compartmentalization of one’s conscience:

At first, I tried to make sense of it. For at least the first 150 days, I kept asking for meaning. Now, I have stopped asking. I have also stopped asking when the world will stop, when the world will stop this genocide. (p.12, W, 22y.o., North Gaza)

This genocide has doubled my age; I see the world differently now. I never imagined we would reach this point, despite the many wars we have lived through, the many explosions and bombings we have survived, and the brutality we have witnessed. But this war has taken away our dreams, our passion, and our loved ones. In the twenty-first century, we die, we starve, we are uprooted, all while the world watches in silence. (p.2, W, 32y.o., displaced to Rafah, Gaza)

Colonial violence aims not only to destroy physically but also to break the spirit and instill a sense of powerlessness that paralyzes any attempt at resistance. At the same time, as this narrative continues, making sense of things, challenging the silence and isolation, and calling events by their proper names was described by Palestinians as a form of resistance, deeply necessary to restore continuity and psychological power:

It is essential to find meaning to survive psychologically. We must resist despair, isolation, and numbness. Colonialism needs us to accept that we can do nothing to make us feel powerless. We must counter it by loudly stating what is happening and why it is happening. (p.2, W, 32y.o., displaced to Rafah, Gaza)

For our research participants, and for us, being able to find communities with whom to share and name and make sense of what is happening helped to ‘piece together the fragments, both personal and those of the dismembered bodies observed in these months’ thus helping us all to reject silence, passivity, and isolation.

## Discussion and Conclusion

The impact of brutal colonial violence on the physical and mental health of the people reached in this work speaks of an intense loss of control and sense, a widespread feeling of emotional numbness and anesthesia, which in turn fuels feelings of guilt and shame, as well as anger and frustration. Palestinians—from Gaza, but also those from the West Bank and the Palestinian diaspora around the world—face the enormous pain of the extermination of their people. They witness the loss of their living spaces, education, and healthcare, the loss of domestic intimacy, already precarious security, and stability. All this destruction undermine the foundations of their daily existence (Kienzler et al., 2024). They experience the loss of relatives and neighbors whom they cannot properly bury, commemorate, or bid farewell to. Experiences to which children are even more vulnerable, exacerbated by the complete absence of safety and extreme daily deprivations of water and food (Boukari et al., 2024). Moreover, this impeded mourning occurs in a broader context that does not recognize the legitimacy of their pain and losses, amplifying community trauma and creating an even deeper sense of isolation and alienation. Recent reports from the Gaza Strip describe adults and children experiencing acute trauma responses, including frozenness, mutism, confusion, attention and memory problems, mood disorders, among others (Save the Children, 2023, 2024). This not only impact the present but creates wounds that are multidimensional and transgenerational (Giacaman, 2018). Palestinians are enduring a second *Nakba*, whose impact has the potential to generate severe intergenerational trauma (Giacaman, 2018; Kienzler et al., 2024).

The impact of colonial and racist violence also reaches those observing Gaza from afar, from countries participating in that oppression, remaining silent, and being ineffective in stopping it. This ineptitude manifests as disorientation, loss of trust, alienation, and isolation. Alongside this psychological crisis of power and connection, the narratives examined here demonstrate continuous attempts to cope with the loss of meaning experienced, producing individual and social suffering.

This work demonstrates how, individually and collectively, people inside and outside Gaza are trying to make sense of and reject the feeling of dehumanization and annihilation (Frankl, 1985). Dehumanization, writes Smith (2014), is not a way of speaking but a way of thinking, acting as a psychological lubricant that dissolves our inhibitions and inflames our destructive passions. It is a fundamental mechanism that allows dominant individuals to project otherness and difference onto the denigrated group (Bhabha, 1999). The colonial self, profiting from the oppression of others, creates a vision of itself that justifies the oppression of the other, who is not seen in their specificity but is instead drawn in anonymous collectivity (Watkins & Shulman, 2010; Memmi, 2013 [1965]). Colonization requires the colonizers to psychologically hold the firm belief that populations forced into a subordinate role are subhuman (Kemp, 2020). Like all sciences, psychological sciences have actively participated and continue to participate in the dehumanization of non-white populations. Precisely because Western-ness and whiteness has a privileged place in our sciences, it is a human and ethical imperative to speak out against and oppose the pervasiveness of racism and the damage it causes. This legacy needs to be known and acknowledged to explain the silence of our institutions. The testimonies collected help us reflect on how dehumanization is not only a tool of violence, but has been necessary—and still is—over these ten months to rationalize one’s detachment and involvement with the images and voices coming from Gaza.

Raz Segal comments, in a recent interview, on how genocide is the culmination of a process that turns the world upside down, transforming defenseless people into dangerous enemies, and violent states into innocent societies threatened by blind hatred and fanaticism and lies into truth. Seen thus, genocide—understood as the destruction of a people and their world—is falsified and rationalized as heroic and just, while resistance to domination is considered a form of madness that needed to be treated (Fanon, 1963) (or exterminated, we might say, looking at the everyday reality). The testimonies clearly show how this entire inversion is deeply present within us and needs to be named and witnessed, though, once again, Palestinians—like all indigenous peoples—seem to be heard as witnesses only through their bloodied bodies. The testimonies highlight a reality where the suffering and loss of Palestinians do not receive the same attention and outrage reserved for other populations in conflict situations. This not only amplifies the pain of the victims but also reinforces a sense of abandonment and isolation. An Italian-Palestinian scholar, Tamara Taher (2023), explains that the word in Arabic for a person who is a witness is Shaheed (شهيد), as in English from the Greek μάρτυς -υρος, meaning “witness”. Jabr (2019) translates it as “one who witnesses with honesty and courage and pays the price.” Palestinians show us day after day that a powerful part of the resistance is a refusal to allow the aggressor to determine the narrative. And, for those of us outside Palestine, what is our role, as we witness this?

As academics and clinicians, it seems to disturb us to engage in these critical self-examinations, especially when politics enters our work and our rooms. We wish we could avoid this topic, and deny our complicity. Yet, the work of solidarity demands engaged witnessing and action. To counter this dehumanization and silencing and to work for the liberation of both the oppressed and the oppressors requires, as Paulo Freire believed, critical self-examination, critical awareness, and, most importantly, collective work, as expressed here by one of our participants outside Gaza:

The other day I was walking through the city where I live, and I was struck by a writing on the wall. It said, ‘The liberation of the Palestinians is needed; it is also a liberation for us.’ I stood there reflecting on it for a while and thought about how true it was. A liberation for us from colonial violence that projects us only into a deadly world, towards being able to build different paths and futures. (29y.o., displaced to Rafah, Gaza)

For those of us who belong to colonizing cultures, working at the intersections of mental health and ongoing violence and oppression, colonial ideologies have contributed to our dissociating the personal from the political, constructing a sense of private interiority strangely disconnected from the historical and cultural context. Our analyses of these narratives in these most unsettling times demonstrate that it is our fundamental task to work alongside each other to reject dehumanization, regain meaning, and bear witness to Palestinian suffering, walking alongside and supporting collective liberation, including our own. Witnessing also means building communities where healing processes can be reactivated, necessarily in relationship with others, as we counteract loneliness, build shared meanings, and transform personal losses into experiences of collective personal and professional significance. Moreover, we believe that internationally, mental health professionals and institutions should engage in critical advocacy, demand accountability for violations of human rights and international law, and support mental health interventions that do not reproduce colonial hierarchies but are instead based on liberation, dignity and care. Academic and clinical institutions in the Global North should reconsider their complicity in knowledge production, and instead promote decolonial alliances and approaches that address structural and political roots of psychological suffering.

To conclude, we want to mention some limitations of this work. Firstly, the number of testimonies collected is relatively small, and the interviews were conducted individually. We plan to further develop this reflection by including group discussions and considerations of possible collective actions. Secondly, the sampling was one of convenience, limited to environments close to the authors. It would be more enriching to explore the opinions of people with much more distant experiences and life contexts to obtain a more complete and perhaps diversified view. Finally, due to word limitations, we are aware that we may have lost some of the richness of the testimonies received.

## Footnotes

1 We use the term genocide in accordance with Raz Segal (director of the genocide studies program at Stockton University) who states that this is *‘a textbook case of genocide*’ and in accordance with the UN Convention on the Prevention and Punishment of the Crime of Genocide defines genocide as “any of the following acts committed with intent to destroy, in whole or in part, a national, ethnical, racial or religious group, as such.” The acts include “killing members of the group, causing serious bodily or mental harm to members of the group, deliberately inflicting on the group conditions of life calculated to bring about its physical destruction in whole or in part, imposing measures intended to prevent births within the group, and/or forcibly transferring children of the group to another group.” (https://www.un.org/en/genocideprevention/documents/atrocity-crimes/). Moreover, we use this term in accordance to many international human rights association (Amnesty International, https://www.amnesty.org/en/documents/mde15/8668/2024/en/; Human Rights Watch, https://www.hrw.org/news/2024/12/19/israels-crime-extermination-acts-genocide-gaza; Doctor without Borders, https://www.doctorswithoutborders.org/latest/life-death-trap-gaza; Oxfam, https://www.oxfam.org.uk/get-involved/campaign-with-oxfam/gaza-israel-crisis-sign-petition-call-for-ceasefire-now/).

2 These numbers, as well as the data presented in this article, refer to August 2024, at the height of the subjugation of labor. As of today, June 2025—613 days since the beginning of the genocide—the figures have drastically increased, and the situation in Gaza has only further deteriorated.

3 This sentence reflects the context in which the article was originally written (August 2024), when the fear of an escalation into genocide was widespread but not yet officially acknowledged. Since then, numerous genocide scholars, legal experts, and human rights organizations have explicitly described the ongoing Israeli assault on Gaza and the broader Palestinian territory as constituting a full-scale genocide.
